# Synergistic difference in the effect of stretching on electromechanical delay components

**DOI:** 10.1371/journal.pone.0300112

**Published:** 2024-03-26

**Authors:** Nicholas Toninelli, Giuseppe Coratella, Stefano Longo, Giulia M. Romani, Christian Doria, Susanna Rampichini, Eloisa Limonta, Fabio Esposito, Emiliano Cè

**Affiliations:** 1 Department of Biomedical Sciences for Health (SCIBIS), Università degli Studi di Milano, Milan, Italy; 2 Division of Exercise Physiology, School of Medicine, West Virginia University, Morgantown, West Virginia, United States of America; 3 IRCSS Galeazzi Orthopedic Institute, Milan, Italy; New Jersey Institute of Technology, UNITED STATES

## Abstract

This study investigated the synergistic difference in the effect of stretching on electromechanical delay (EMD) and its components, using a simultaneous recording of electromyographic, mechanomyographic, and force signals. Twenty-six healthy men underwent plantar flexors passive stretching. Before and after stretching, the electrochemical and mechanical components of the EMD and the relaxation EMD (R-EMD) were calculated in gastrocnemius medialis (GM), lateralis (GL) and soleus (SOL) during a supramaximal motor point stimulation. Additionally, joint passive stiffness was assessed. At baseline, the mechanical components of EMD and R-EMD were longer in GM and GL than SOL (Cohen’s *d* from 1.78 to 3.67). Stretching decreased joint passive stiffness [-22(8)%, *d* = -1.96] while overall lengthened the electrochemical and mechanical EMD. The mechanical R-EMD components were affected more in GM [21(2)%] and GL [22(2)%] than SOL [12(1)%], with *d* ranging from 0.63 to 1.81. Negative correlations between joint passive stiffness with EMD and R-EMD mechanical components were found before and after stretching in all muscles (*r* from -0.477 to -0.926; *P* from 0.007 to <0.001). These results suggest that stretching plantar flexors affected GM and GL more than SOL. Future research should calculate EMD and R-EMD to further investigate the mechanical adaptations induced by passive stretching in synergistic muscles.

## Introduction

Synergistic muscles act together to generate movements in different directions. Changes in the mechanical properties of one of these muscles can result in alterations of overall force production, potentially compromising performance [1] and elevating the risk of injury [2]. Various approaches have been employed to assess the mechanical properties of synergistic muscles, primarily through indirect methods such as B-mode ultrasonography [3, 4] or shear wave elastography [5–8]. The electromechanical delay (EMD) and the electromechanical delay during relaxation (R-EMD) represent the time lag between the onset of electromyographic (sEMG) and force signals during contraction [9] and the duration between the cessation of sEMG activity and the decaying of force during relaxation [10], respectively. The conventional assessment method combines concurrent recording of sEMG and force during isometric contractions, capturing EMD and R-EMD comprehensively without distinction between their electrochemical and mechanical components [9–11]. A recent advancement involving the incorporation of the mechanomyogram (MMG) in EMD and R-EMD calculation, has addressed this issue, potentially serving as a useful tool to assess the properties of synergistic muscles [12–15]. This approach enables the subdivision of EMD into: (i) a mainly electrochemical component, involving the time between the onset of the sEMG to the MMG signal (Δt EMG-MMG), and (ii) a mainly mechanical component, providing the time between the onset of the MMG and the onset of the force development (Δt MMG-F) [12, 13, 16–19]. Similarly, the R-EMD is divided into an initiating electrochemical component, from the end of the EMG signal to the beginning of force decay (R-Δt EMG-F), possibly including the beginning of Ca^2+^ re-uptake and the cross-bridges transition from a force-generating to a non-force generating condition, and three consecutive primarily mechanical components, representing events from cross-bridge detachment to the return to a pre-contraction state: i) the initial force decay to the beginning of the largest MMG displacement (R-Δt F-MMG); ii) the largest MMG displacement duration (R-Δt MMG_p-p_); and iii) the time lag from the end of the largest MMG displacement to the return to the baseline of the force signal (R-Δt MMG-F_END_) [13–15, 20]. Previous studies have reported an increase in both electrochemical and mechanical components of EMD and R-EMD after passive stretching [12, 13, 17, 19, 21]. Interestingly, negative correlations between the joint passive stiffness and the duration of the mechanical but not electrochemical components calculated in *gastrocnemius medialis* (GM) were reported before and immediately after a passive stretching bout [13]. However, the approaches used in those studies did not allow for the simultaneous examination of potential heterogeneous differences in the stretch-induced effects on EMD and R-EMD between plantar flexor synergists. Biarticular muscles might experience larger decreases in stiffness (hardness) than monoarticular muscles after passive stretching [6–8, 22, 23]. Differences in morphological characteristics [6, 24], angle of insertion of the muscle fascicles into the tendon [5, 6, 25], or initial slack angle, i.e., the joint angle beyond which muscles begin to develop passive tension [5, 6, 25] were advocated as possible factors accounting for the differences in stiffness or hardness found at rest or in response to interventions between biarticular and monoarticular muscles. These possible between-muscle differences may affect both the joint passive stiffness and the mechanical components of the EMD and R-EMD in synergistic muscles, thus altering the correlation between the two variables. However, none of the previous studies attempted to determine such a correlation. On these bases, by adding MMG to EMD and R-EMD calculation, we aimed to: i) assess possible between-muscle differences in the effects of a passive stretching bout on the EMD and R-EMD components in synergistic muscles; and ii) determine the correlations between the joint passive stiffness and the EMD and R-EMD mechanical components in the different synergistic muscles, both before and after stretching.

## Methods

### Study design

The present study was conceived as a pre-post, within-subject, cross-sectional study. Passive stretching was applied to the plantar flexors, and the synergistic muscles were identified as GM, gastrocnemius lateralis (GL) and soleus (SOL). Based on a previous investigation [13], we used the stretch-induced changes in the Δt MMG-F as the main outcomes (Cohen’s *d* = 0.84), a three-way analysis of variance for repeated measures (*session* [two-levels]: stretching, control; *time* [two-levels]: PRE, POST; and *muscle* [three levels]: GM, GL, and SOL) as statistical model, an α = 0.05, a 1-β err = 0.80, a correlation between the repeated measures = 0.7, and non-sphericity correction = 1 to calculate the sample size using statistical software (G-Power 3.1, Dusseldorf, Germany). The resulting sample was 21 participants. Moreover, from preliminary values calculated on a subsample of 12 participants from the correlation between Δt MMG-F of the three muscles and the joint passive stiffness, using a correlation bivariate model, two tails, correlation ρH1 = 0.866, correlation ρH0 = 0.600, α = 0.05, 1-β err = 0.80, the required sample size resulted in 24 participants.

### Participants

Twenty-six men [age: 23(3) yrs.; body mass: 74(2) kg; stature: 1.79(0.05) m; mean (standard deviation)] took part in this study. The inclusion criteria were: i) no clear orthopaedical and/or neurological pathologies, ii) no lower-limb muscular or joint injury in the previous 6 months, and iii) no involvement in a systematic passive stretching training. The local University Ethics Committee approved the study (*CE*84/23) that was performed following the principles of the latest version of the Declaration of Helsinki. The participants gave their written informed consent after a full explanation on the purpose of the study and the experimental design. On the test days, participants came to the laboratory after fasting overnight, abstaining from caffeine and other similar substances for at least 12 h, and not taking part in heavy exercise for at least 48 h before the tests. They were free to withdraw from the study at any time.

### Procedures

All measurements were performed in a laboratory with constant room temperature [20(2°C)] and humidity [50(3%)]. To minimize the circadian changes in force and joint mobility, the tests were conducted at the same hour between 9:00 AM and 12.00 noon. The participants visited the laboratory four times. During the first two sessions, they were familiarized with the experimental set-up, getting accustomed to the motor point stimulation and the procedure to define the joint passive stiffness. On this occasion, a map with some identification points over the skin (moles, scars, angiomas), together with the position of the angle transducers, sEMG electrode arrays, and accelerometers were drawn on transparency sheets to allow accurate electrodes repositioning consistency within the same area. In this session, the participants were accustomed to the discomfort induced by the passive stretching through a visual analogue scale. Moreover, during the second session the between-muscle crosstalk was calculated. The third and fourth sessions were proposed in a randomized order for i) a unilateral passive stretching, or ii) the control session (CTRL). Within the third and fourth session, the ankle range of motion (ROM) and the joint passive stiffness were firstly defined. Second, the tetanic torque of GM, GL, and SOL was assessed. During this procedure, the sEMG, MMG and force signals were detected on GM, GL, and SOL, allowing the identification of the EMD and R-EMD components for each muscle. All measurements were performed in the dominant limb before and after a unilateral passive stretching bout involving the plantar flexors.

### Measurements

#### Between-muscle crosstalk

**Fig 1:**
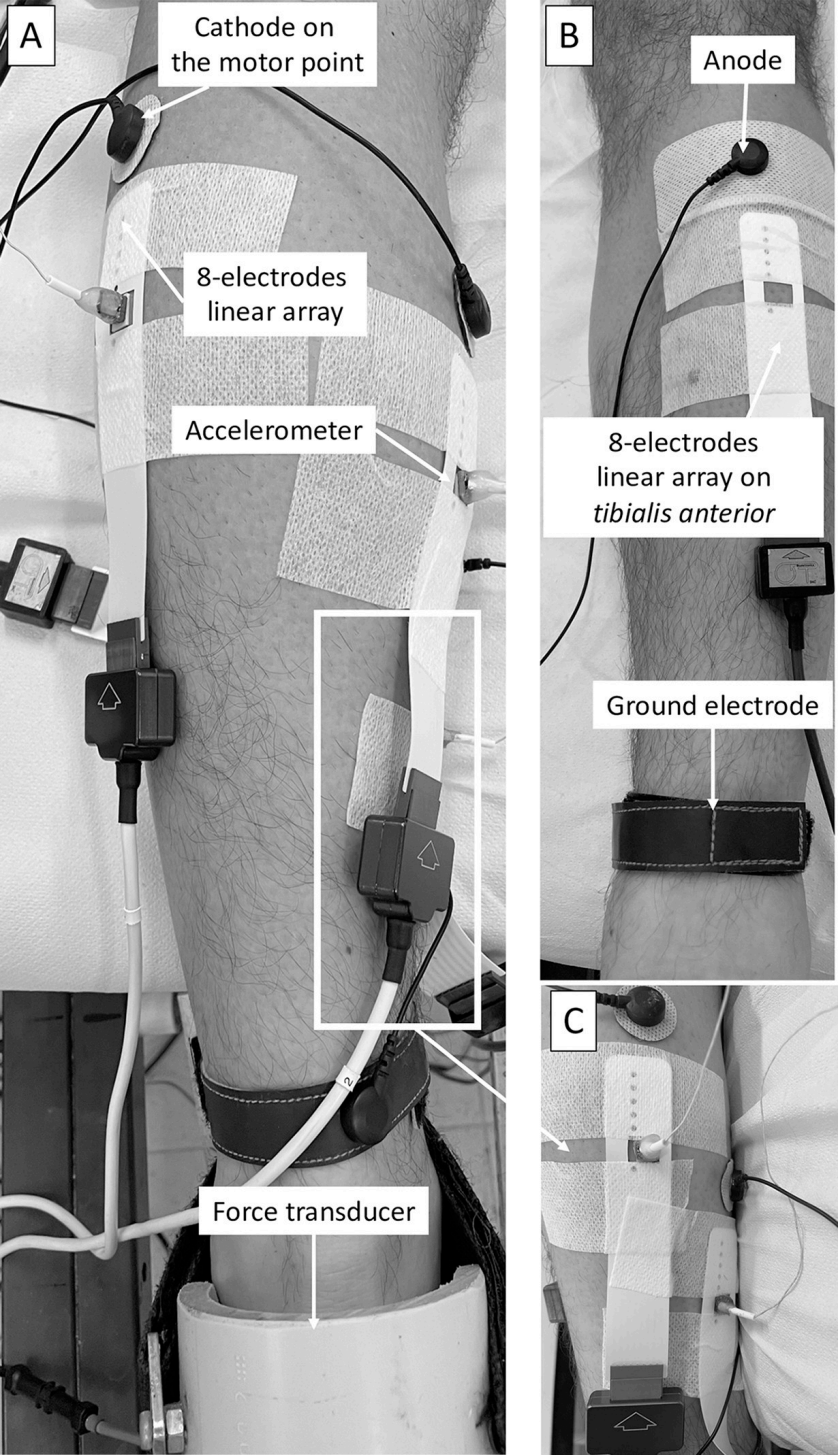
Experimental setup. Accelerometers and surface electromyographic 8-channel linear arrays on the synergistic muscles *gastrocnemius medialis*, *gastrocnemius lateralis* (panel A) and *soleus* (panel C), and location of the 8-channel linear array on the *tibialis anterior* (antagonist muscle) (panel B). Load cell and anode and cathodes electrodes position is provided.

Fig 1 represents the sEMG and MMG electrodes positioning. During the second session, the between-muscle crosstalk in the synergistic (GM, GL, and SOL) and in the antagonist muscle (*tibialis anterior*) was evaluated. The participants laid prone, with the knee fully extended and the tested ankle at a neutral position (0°). The ankle was firmly secured with a Velcro® strap (Velcro Industries Inc., Willemstad, Netherlands Antilles). After cleaning the skin with ethyl alcohol, the main motor point of GM, GL, SOL was localized by a pen electrode to determine the cathode position (8 mm diameter; Medicompex SA, Ecublens, Switzerland) of the stimulator (Digitimer Stimulator Model DS7AH, Hertfordshire, UK; stimulation characteristics: pulse 1 ms, with an inter-pulse duration of 10 ms). A common anode (50 × 100 mm rectangular electrode; Medicompex SA, Ecublens, Switzerland), was placed anteriorly at the proximal third of the leg. The sEMG and MMG signals were detected on each muscle by a linear array of eight electrodes (mod. ELSCH008, OtBioelettronica, Turin, Italy; 125 × 25 mm; electrode length 2 mm; inter-electrode distance 5 mm) and by a monodirectional accelerometer [model ADXL103; Analog Devices, Norwood, MA, USA; device mass <1.0 g; sensitivity 1000 mV·g^−1^; measure range (1.7 g)]. Another linear array of eight electrodes was placed on the *tibialis anterior*. For each muscle, the stimulation amplitude generating the maximum M-wave was assessed with +5 mA steps starting from 30 mA. Such a stimulation current was then increased by +10% during the assessment procedures. After 20 min of passive recovery, the stimulation was evoked individually on each muscle [mean stimulation current: GM = 107(15) mA; GL = 93(12) mA; SOL = 91(9) mA], and the M-wave and MMG_p-p_ signals generated by the stimulation on the synergistic and antagonist muscle (M-wave only) were recorded. Three stimulations *per* muscle were elicited, with 3 min of passive recovery between each stimulation. The order of the muscle stimulated was randomized. For each muscle, the peak-to-peak of both M-wave and MMG signal from the muscle directly stimulated was first calculated and used to normalize the amplitude of the signal elicited during the stimulations of the other muscles.

#### Dorsiflexion ROM

The ankle was securely fixed to a custom-made ergometer for assessing the dorsiflexion ROM and the joint passive stiffness. A previously calibrated bi-axial angle transducer (mod. TSD 130A, Biopac System, CA, USA) was positioned on the external face of the fibula and on the calcaneum to monitor the changes in ankle ROM. After 10 passive ankle movements performed by an operator, the dorsiflexion ROM was determined starting with the ankle at its neutral position (~0° of dorsiflexion) [26], and manually slowly dorsiflexed to avoid the activation of any muscle reflex, as monitored by the sEMG signal, until the maximum point of discomfort was reached. The maneuver was performed with the participant lying prone on an experimental bed, with the knee fully extended. The difference between the ankle neutral position and the angle at the point of discomfort was considered the joint ROM [27].

#### Joint passive stiffness

After the ROM assessment, with the participant remaining in a prone position on the experimental bed and with the knee fully extended, the same ergometer was manually fixed at 0°, 10°, 20° of ankle dorsiflexion, and at end ROM [28] to allow measurements of passive force. The mobile metal plate was connected to a previously calibrated load cell (mod. SM-2000 N, Interface, UK; operating linearly between 0 and 2000 N). Joint positioning was executed at a slow speed to avoid reflex muscle activation (monitored by sEMG signal). The passive force exerted by the plantar flexors was recorded at each angle as the average passive force during the first 5 s after ankle positioning. This short time period allowed the operator to minimize the influence of the static position on the joint viscoelastic properties [28]. The passive force signal was acquired by A/D converter (mod. UM 150 Biopac; Biopac System Inc.), sampled at 2000 Hz, and driven to a multichannel amplifier (mod. EMG-USB, OtBioelettronica, Turin, Italy). The passive force-angle curve between 0° and 20° of dorsiflexion was fitted with the best polynomial regression model [21, 29], and the slope of this curve at 20° of dorsiflexion represented the joint passive stiffness. In participants not reaching the 20° of dorsiflexion, the maximum dorsiflexion angle reached before stretching was used to build the force-angle curve. The slope of the curve at the maximum angle was used as joint passive stiffness.

#### Tetanic stimulation

The participants were positioned in the same ergometer and were equipped as in the between-muscle crosstalk procedure, with the ankle at a neutral position (0°) and the knee joint fully extended. After the identification of stimulus that elicited the maximal M-wave, the participants rested for 5 min. Thereafter, three tetanic stimulations (one stimulation *per* each muscle), consisting of a train of pulses (wave shape: biphasic; pulse duration: 300 μs; stimulation frequency: 50 Hz; current amplitude: +10% of the maximum M-wave; duration: 2 s) were delivered, with 5 s of pause between each stimulation. The order of the muscle stimulated was randomized. During the stimulations, the participants were instructed to maintain the muscles as relaxed as possible. The lack of activation of the *tibialis anterior* was checked during contraction by linear arrays of eight electrodes (mod. ELSCH008, OtBioelettronica, Turin, Italy; 125 × 25 mm; electrode length 2 mm; inter-electrode distance 5 mm).

#### sEMG and MMG

The sEMG and MMG signals were detected from GM, GL and SOL and acquired by a multichannel amplifier (mod. EMG-USB, OtBioelettronica, Turin, Italy; input impedance: >90 MX; CMRR: >96 dB; sEMG and MMG filter type: IV order Butterworth filter, bandwidth: 10–500 and 4–120 Hz, respectively; gain: × 1000 and × 20 for sEMG and MMG, respectively), with a sampling rate of 10,240 Hz. The sEMG signal was detected by three linear arrays of eight electrodes (mod. ELSCH008, OtBioelettronica, Turin, Italy; 125 × 25 mm; electrode length 2 mm; inter-electrode distance 5 mm) fixed to the skin by dual-adhesive foam (mod. AD008, OtBioelettronica, Turin, Italy) and filled with conductive gel (Cogel, Comedical, Trento, Italy). The skin area under the sEMG electrodes was cleaned with ethyl alcohol, abraded gently with fine sandpaper, and prepared with a conductive cream (Nuprep, Weaver and Co., Aurora, USA) to achieve an inter-electrode impedance below 2000 Ω. The third electrode of the sEMG array was removed and replaced by a mono-directional accelerometer (mod. ADXL103, Analog Devices, Norwood, MA, USA; device mass: <1.0 g; sensitivity: 1000 mV/g; measure range: ± 1.7 g) for the MMG signal detection, from the same muscle area as sEMG. The accelerometer provided a measurement of the acceleration on the *y*-axis occurring during muscle contraction. The sEMG arrays were positioned parallel to the major axes of the fibers between the tendon and the motor point, in accordance with the European recommendations for surface EMG [30, 31]. A reference electrode was placed around the ankle [32].

### Data analysis

The tetanic torque was obtained by multiplying the peak tetanic force generated during the stimulation for each muscle by the distance between the apical aspect of the external malleolus and the force application point.

#### Delays calculation

Similar to previous investigation the EMD and R-EMD components were calculated as follows [30, 31]. EMD was divided into i) Δt EMG-MMG (mainly electrochemical component), and (ii) Δt MMG-F (mainly mechanical component). Δt EMG-MMG was calculated from the first positive deflection of the M wave to the onset of the MMG signal. Similarly, Δt MMG-F was calculated as the time lag between MMG signal onset and the force signal. EMD was considered as the sum of the two components. Three standard deviations from the mean baseline noise measured over a time window of 200 ms were set for detecting the onset of each signal. R-EMD was partitioned into four components: i) R-Δt EMG-F (mainly electrochemical component) spanning from sEMG cessation to the beginning of force decay; ii) R-Δt F-MMG (first mainly mechanical component) from the beginning of force decay to the beginning of the R-MMG complex (the last negative peak before the development of the largest MMG displacement); iii) R-Δt MMG_p-p_ (second mainly mechanical component) from the beginning to the end of the R-MMG complex (defined as the return of the MMG signal to baseline); and iv) R-Δt MMG-F_END_ (third mainly mechanical component) from the end of the R-MMG complex to the return to baseline of force. Three standard deviations from the mean baseline noise measured over a time window of 200 ms were set for detecting the beginning of force decay, the end of the R-MMG complex, and the return of force to baseline.

### Passive stretching

During the passive stretching protocol, the participants remained prone on the same ergometer used for the testing procedures, with the knee joint fully extended. As in a previous study [33], an operator dorsiflexed the ankle of the stretched limb until 90% of maximal discomfort, according to the subjective response of each participant (0–10 visual analogue scale: 0 = no discomfort, 10 = maximal discomfort, level of perceived discomfort required = 9). The force output between the passively stretched leg and the operator’s arms was recorded during the protocol by a load cell (SM-2000 N, Interface, Crowthorne, UK) [33]. Specifically, the load cell was positioned 5 cm above the metatarsus of the stretched limb and an operator pushed perpendicularly to the load cell to stretch the plantar flexors. To minimize any possible muscle reflex activity, the muscle elongation was reached in 6 s and maintained for 45 s [34]. In line with previous investigations, five 45-s sets with 15 s intervals of passive recovery were performed for a total stretching duration of 225 s [17, 21, 33]. The sEMG signal was checked during passive stretching to monitor any possible muscle activation during the elongation [17]. In the CTRL session, the participants laid prone as relaxed as possible with the ankle at a neutral angle (0°) for an equivalent duration.

### Statistical analysis

Statistical analysis was performed using a statistical software package (IBM SPSS Statistics 27, Armonk, NY). The Shapiro–Wilk’s and Mauchly’s tests checked the normal distribution and the sphericity of the sampling, respectively. Greenhouse‐Geisser correction was performed if the sphericity assumption was violated. The measurements taken during the first two sessions were utilized to calculate intersession reliability and sensitivity. Reliability was calculated with a two-way random, consistency type intraclass correlation coefficient (ICC). Cronbach’s α was classified as: very high (≥0.90); high (0.89 to 0.70); moderate (0.69 to 0.50) and the percentage standard error of the measurement (SEM%) was calculated. The minimum detectable change with a 95% confidence interval (MDC_95%_) defined sensitivity. The pre-post difference in ROM, tetanic torque, and joint passive stiffness was determined between stretching and control by a two-way (*session* × *time*) analysis of variance (ANOVA) for repeated measures. To calculate the between-muscle (GM, GL, and SOL) differences in the sEMG and MMG parameters and in the different delay components, a three-way (*session* × *time* × *muscle*) ANOVA for repeated measures was performed. The significance level was set at *P*-value of 0.05 but adjusted using the Bonferroni correction where appropriate. If not otherwise stated, descriptive statistics are presented as mean (SD) or 95% confidence interval (CI_95%_). The magnitude of the interactions and main effects was calculated using partial eta squared (η_p_^2^), interpreted as small (0.01–0.059), medium (0.06–0.139), and large (≥0.14). The magnitude of the pairwise comparisons was determined using Cohen’s *d* interpreted as trivial (0–0.19), small (0.20–0.59), moderate (0.60–1.19), large (1.20–1.99) and very large (≥2.00) [35]. Pearson’s product moment correlation coefficient (*r*) was used to determine the correlations between the different delay components and the joint passive stiffness and interpreted as trivial <0.1, low (0.11–0.29), moderate (0.30–0.49), high (0.50–0.69), very high (0.70–0.89), nearly perfect (0.90–0.99), and perfect (= 1.00) [35].

## Results

### Reliability

Table 1 presents the reliability (ICC and SEM%) and the sensitivity variables (MDC_95%_). The ICC ranged from 0.901 to 0.998 and the SEM% from 0.32% to 4.48%. MDC_95%_ ranged from 0.63% to 8.79%.

**Table 1:** Intersession reliability [ICC with its 95% confidence interval (CI_95%_) and SEM%] and sensitivity (MDC_95%_) for each dependent parameter.

			Trial 1 [mean (SD)]		Trial 2 [mean (SD)]		ICC (95% CI)			SEM (%)	MDC_95%_ (%)
Tetanic torque (Nm)		GM	77	16	77	16	0.997	0.992	0.998	1.12	2.19
		GL	79	17	79	17	0.957	0.906	0.980	4.48	8.79
		SOL	67	17	67	17	0.982	0.960	0.992	3.46	6.78
EMD (ms)		GM	26.6	2.4	26.3	2.6	0.974	0.801	0.994	1.52	2.99
		GL	26.8	2.5	26.4	2.3	0.977	0.812	0.995	1.37	2.68
		SOL	23.5	2.5	23.4	2.4	0.907	0.805	0.957	3.16	6.19
Δt EMG-MMG (ms)		GM	9.7	1.0	9.7	1.2	0.919	0.702	0.959	3.34	6.54
		GL	9.6	1.2	9.3	1.0	0.944	0.661	0.986	2.75	5.38
		SOL	9.7	1.0	9.8	1.0	0.995	0.611	0.999	0.75	1.48
Δt MMG-F (ms)		GM	16.9	2.0	16.6	2.0	0.989	0.807	0.998	1.27	2.50
		GL	17.2	2.0	17.0	2.0	0.996	0.821	0.999	0.73	1.43
		SOL	13.8	2.1	13.6	2.0	0.967	0.727	0.998	2.74	5.37
R-EMD (ms)		GM	243	18	240	18	0.986	0.896	0.997	0.88	1.73
		GL	242	19	241	19	0.997	0.737	0.999	0.43	0.84
		SOL	232	18	231	18	0.994	0.985	0.997	0.60	1.17
R-Δt EMG-F (ms)		GM	19.7	3.1	19.9	3.3	0.985	0.612	0.997	1.97	3.86
		GL	19.6	3.2	19.0	3.1	0.980	0.732	0.995	2.31	4.52
		SOL	19.7	3.1	19.9	3.1	0.998	0.888	0.999	0.70	1.37
R-Δt F-MMG (ms)		GM	45.2	2.1	44.7	2.1	0.978	0.844	0.996	0.71	1.38
		GL	45.4	2.6	45.1	2.9	0.980	0.903	0.993	0.85	1.67
		SOL	40.9	2.4	40.5	2.2	0.901	0.709	0.985	1.76	3.45
R-Δt MMG_p-p_ (ms)		GM	101	7	101	7	0.998	0.769	0.999	0.32	0.63
		GL	102	7	101	7	0.993	0.984	0,997	0.60	1.17
		SOL	98	7	98	7	0.997	0.992	1.000	0.40	0.78
R-Δt MMG-F_END_ (ms)		GM	76.3	7.8	75.9	7.7	0.997	0.984	0.999	0.56	1.09
		GL	75.6	8.4	75.4	8.4	0.998	0.986	1.000	0.50	0.97
		SOL	72.8	7.9	72.5	8.0	0.995	0.988	0.998	0.78	1.52

ICC denotes intraclass correlation coefficient; SEM%, percentage standard error of measurement; MDC_95%_, minimum detectable change with a 95% confidence interval. EMD and R-EMD, total delay during contraction and relaxation phases, respectively; Δt EMG-MMG and R-Δt EMG-F, mainly electrochemical components of EMD and R-EMD, respectively (see text for details); Δt MMG-F, mainly mechanical EMD component; R-Δt F-MMG, R-Δt MMG_p-p_, and R-Δt MMG-F_END_, R-EMD mainly mechanical components (see text for details); GM, *gastrocnemius medialis*; GL, *gastrocnemius lateralis*; SOL, s*oleus*.

### Between-muscle crosstalk

Table 2 reports a crosstalk of the peak-to-peak MMG signal ranging from 6(1)% to 10(3)% and crosstalk of the M-wave spanning from 9(5)% to 11(7)% in the synergistic muscles and from 5(3)% to 6(3)% in the *tibialis anterior*.

**Table 2:** Between-muscle crosstalk.

	Stimulated muscle	Crosstalk							
	MMG_p-p_ (m∙s^-2^)	GM		GL		SOL			
		MMG_p-p_ (m∙s^-2^)	%	MMG_p-p_ (m∙s^-2^)	%	MMG_p-p_ (m∙s^-2^)	%		
GM	14.8(1.2)	---	---	0.8(0.1)	6(1)	0.9(0.2)	6(2)		
GL	14.9(3.9)	1.3(0.3)	9(4)	---	---	1.4(0.3)	10(3)		
SOL	15.0(3.9)	1.4(0.3)	10(3)	1.3(0.4)	9(3)	---	---		
	Stimulated muscle	Crosstalk							
	M-wave (mV)	GM		GL		SOL		TA	
		M-wave (mV)	%	M-wave (mV)	%	M-wave (mV)	%	M-wave (mV)	%
GM	4.1(1.7)	---	---	0.4(0.4)	11(7)	0.4(0.2)	9(5)	0.2(0.2)	6(3)
GL	3.3(1.2)	0.4(0.2)	11(5)	---	---	0.3(0.3)	9(5)	0.2(0.1)	6(3)
SOL	2.2(0.7)	0.2(0.2)	10(8)	0.2(0.2)	10(6)	---	---	0.1(0.1)	5(3)

MMG, mechanomyogram; p-p_,_ peak-to-peak elicited during stimulation; GM, *gastrocnemius medialis*; GL, *gastrocnemius lateralis*; SOL, *soleus*; TA, *tibialis anterior*. Data presented as mean(SD).

### ROM and joint passive stiffness

**Fig 2:**
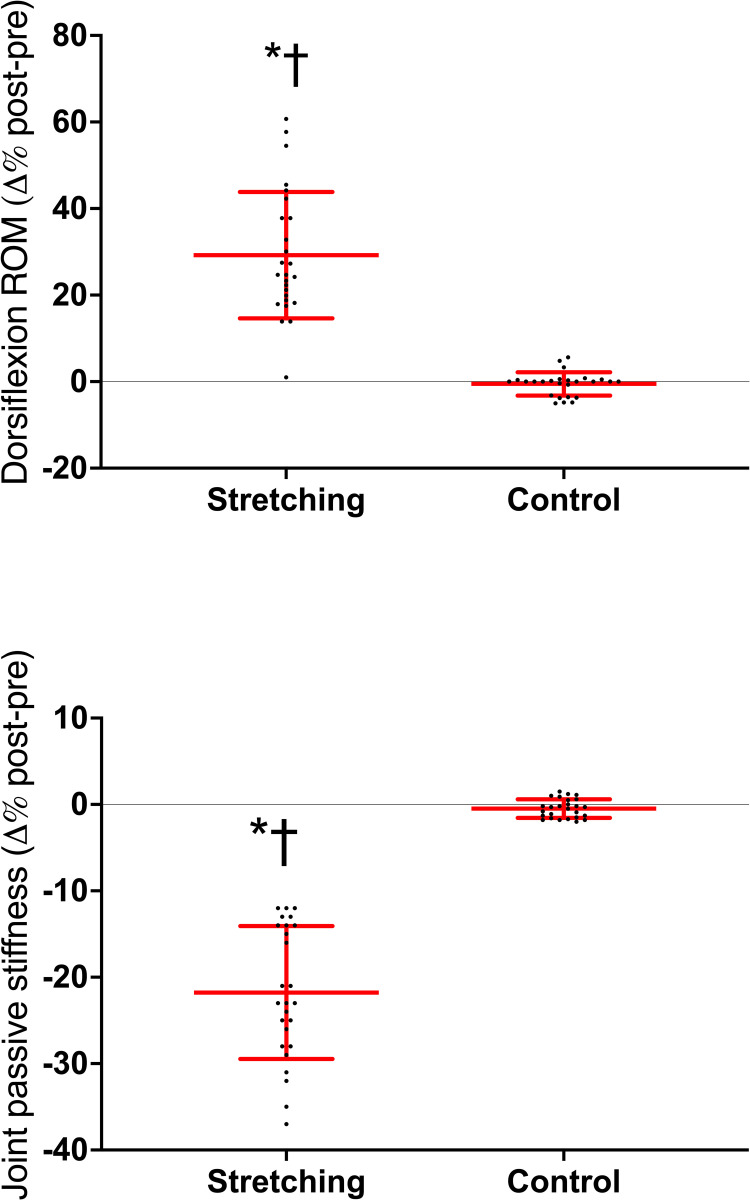
Individual and average post-pre percentage changes in the ankle dorsiflexion range of motion (ROM) and joint passive stiffness. *P<0.05 post *vs*. PRE; †*P*<0.05 *vs*. control. Horizontal red line represents the average values, the vertical lines represent the standard deviation.

The stretch-induced changes in dorsiflexion ROM and joint passive stiffness are shown in Fig 2. Two-way ANOVA showed *session* × *time* interaction for ROM (*F*_*1*_ = 27.91, *P*<0.001, η_p_^2^ = 0.736) and joint passive stiffness (*F*_*1*_ = 16.22, *P*<0.001, η_p_^2^ = 0.538). At POST, the dorsiflexion ROM increased [29(15) %, *d* = 1.18 (0.58/1.75)], while joint passive stiffness decreased in the stretched limb [22(8) %, *d* = -1.96 (-2.63/-1.30)]. No change occurred in CTRL.

### Tetanic torque

**Fig 3:**
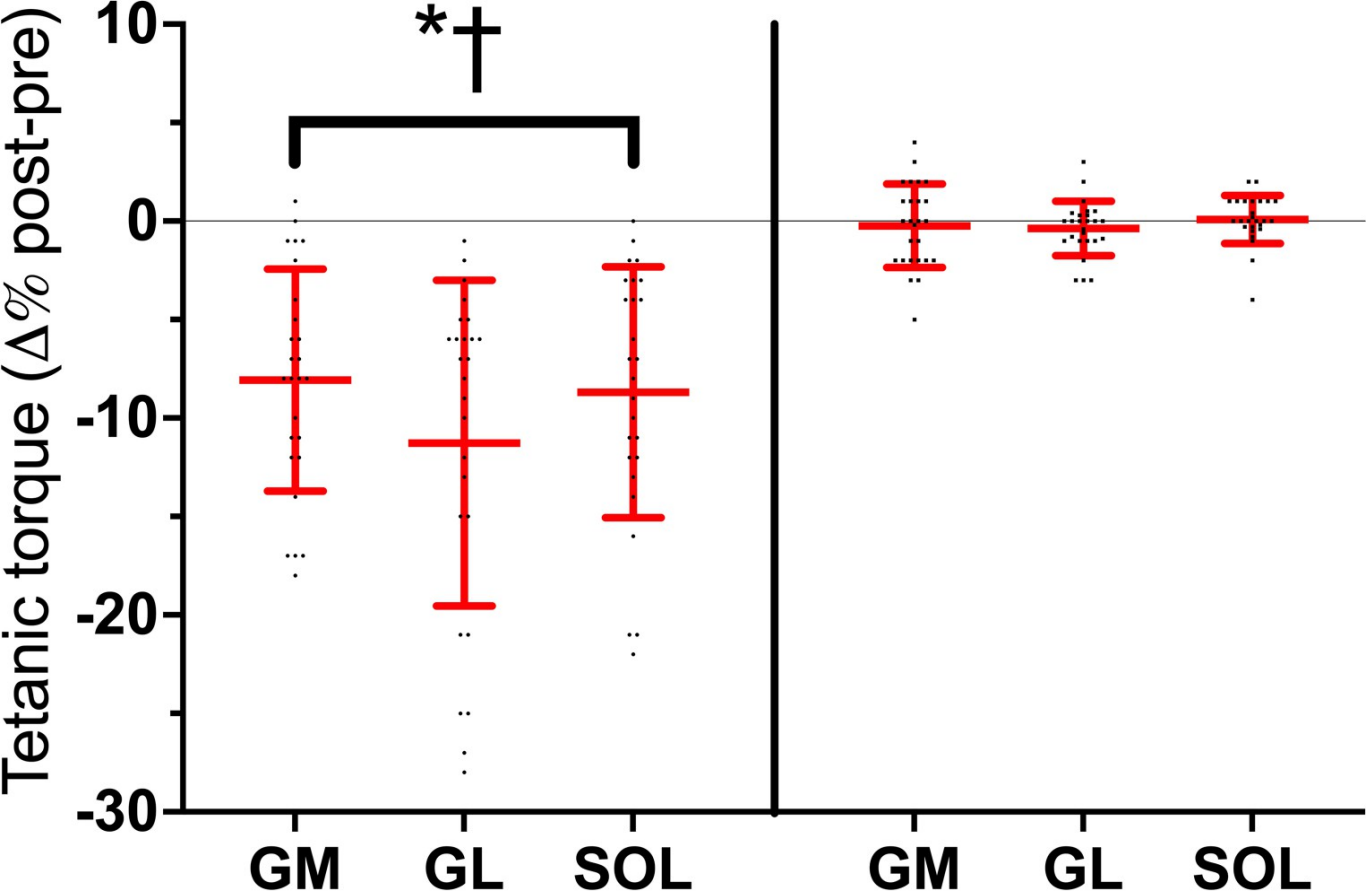
Individual and average post-pre percentage changes in the tetanic torque after passive stretching and control in the *gastrocnemius medialis* (GM), *gastrocnemius lateralis* (GL) and *soleus* (SOL). ● = stretched limb; ■ = control; *P<0.05 post *vs*. PRE; †*P*<0.05 *vs*. control. Horizontal red line represents the average values, the vertical lines represent the standard deviation.

Fig 3 shows the stretch-induced changes in tetanic torque. Three-way ANOVA found *session* × *time* interaction (*F*_*1*_ = 14.63, *P* = 0.001, η_p_^2^ = 0.309). The tetanic torque decreased to a similar extent in GM, GL, and SOL by ~9(7) %, [(*d* from -1.49 to -1.24)].

### EMD and R-EMD

**Fig 4:**
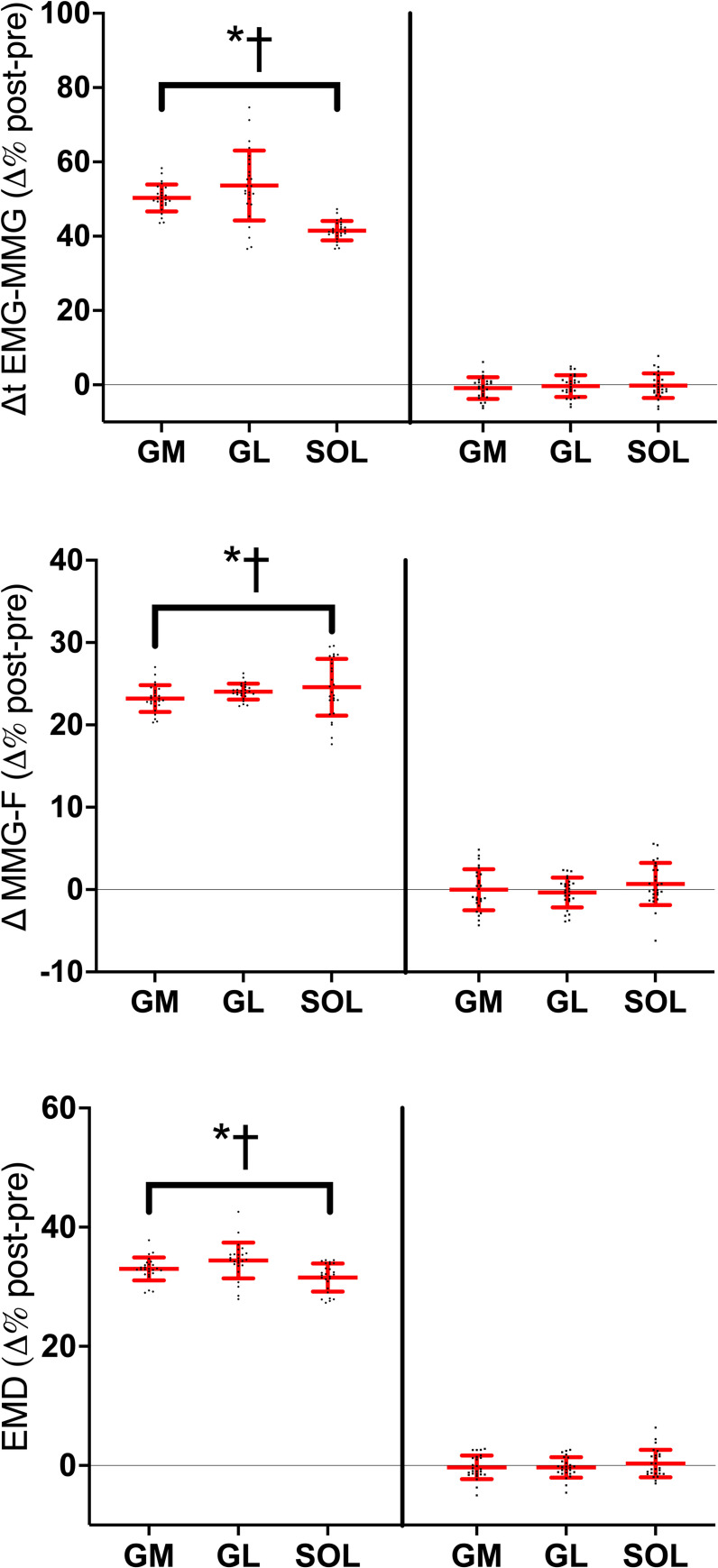
Individual and average post-pre percentage changes in the electromechanical delay (EMD) components after passive stretching and control in the *gastrocnemius medialis* (GM), *gastrocnemius lateralis* (GL) and *soleus* (SOL). ● = stretched limb; ■ = control; *P<0.05 post *vs*. PRE; †*P*<0.05 *vs*. control. Horizontal red line represents the average values, the vertical red lines represent the standard deviation. EMD, total delay during contraction phase; Δt EMG-MMG, mainly electrochemical component of EMD; Δt MMG-F, mainly mechanical EMD component; see text for details on each component.

Absolute means and standard deviations in EMD and R-EMD components are summarized in Table 3. The stretch-induced changes in the EMD are provided in Fig 4. Three-way ANOVA found *session* × *time* × *muscle* interaction in Δt EMG-MMG (*F*_*2*_ = 6.50, *P*<0.001, η_P_^2^ = 0.446),Δt MMG-F (*F*_*1*,*2*_ = 10.32, *P*<0.001, η_P_^2^ = 0.408), and EMD (*F*_*1*,*2*_ = 9.19, *P*<0.001, η_P_^2^ = 0.135). At baseline, the average between-muscle EMD [26.3(2.4) ms and 26.6(2.5) ms vs. 23.5(2.5) ms, in GM, GL, and SOL, respectively] and Δt MMG-F [16.7(2.1) ms and 17.1(2.0) ms vs. 13.7(2.1) ms, in GM, GL, and SOL, respectively], were longer in GM and GL than in SOL (*d* from 1.78 to 2.11). No between-muscle differences were observed in Δt EMG-MMG. Stretching increased all the EMD components similarly in all muscles (*d* from 0.12 to 4.58). No change occurred in CTRL.

**Table 3:** Absolute mean and standard deviation in electromechanical delay (EMD) and electromechanical delay during relaxation (R-EMD) components.

		CTRL			STR		
		GM	GL	SOL	GM	GL	SOL
Δt EMG-MMG (ms)	PRE	8.7(1.2)	9.3(1.1)	9.8(1.1)	9.7(1.1)	9.5(1.2)	9.7(1.1)
	POST	8.6(1.2)	9.3(1.1)	9.8(1.2)	14.5(1.2)	14.5(0.9)	13.7(1.3)
Δt MMG-F (ms)	PRE	16.7(2.0)	17.1(2.0)	13.7(1.9)	16.7(2.1)	17.1(2.0)	13.7(2.1)
	POST	16.7(2.0)	17.0(2.0)	13.8(1.9)	20.5(2.3)	21.2(2.3)	17.1(2.6)
EMD (ms)	PRE	25.4(2.5)	26.4(2.4)	23.5(2.2)	26.3(2.4)	26.6(2.5)	23.5(2.5)
	POST	25.3(2.5)	26.3(2.4)	23.6(2.3)	35.0(2.7)	35.7(2.6)	30.8(3.0)
R-Δt EMG-F (ms)	PRE	18.7(3.2)	19.1(3.2)	19.8(3.2)	18.8(3.2)	19.3(3.1)	19.8(3.1)
	POST	18.8(3.2)	19.0(3.2)	19.8(3.3)	21.3(3.6)	21.9(3.6)	22.4(3.6)
R-Δt F-MMG (ms)	PRE	44.7(2.1)	45.1(2.6)	40.7(2.4)	44.9(2.1)	45.3(2.7)	40.7(2.2)
	POST	44.6(2.2)	45.0(2.6)	40.8(2.4)	55.3(2.4)	56.3(3.4)	45.5(2.4)
R-Δt MMG_p-p_ (ms)	PRE	101.0(7.2)	101.4(7.2)	98.2(7.2)	101.2(7.2)	101.5(7.2)	98.3(7.1)
	POST	101.3(7.3)	101.6(7.1)	98.3(7.3)	106.3(7.4)	106.3(7.6)	103.5(7.9)
R-Δt MMG-F_END_ (ms)	PRE	76.0(7.8)	75.3(8.4)	72.6(7.9)	76.1(7.7)	75.5(8.4)	72.6(8.0)
	POST	76.1(8.0)	75.4(8.6)	72.9(8.0)	109.6(7.7)	108.8(10.1)	86.6(7.9)
R-EMD (ms)	PRE	240.4(17.9)	240.9(18.9)	231.4(18.2)	241.1(17.9)	241.5(18.8)	231.4(17.8)
	POST	240.8(18.2)	241.1(18.9)	231.7(18.5)	292.5(18.1)	293.3(20.3)	258.0(18.4)

CTRL, control condition; STR, passive stretching condition; EMD and R-EMD, total delay during contraction and relaxation phases, respectively; Δt EMG-MMG and R-Δt EMG-F, mainly electrochemical components of EMD and R-EMD, respectively (see text for details); Δt MMG-F, mainly mechanical EMD component; R-Δt F-MMG, R-Δt MMG_p-p_, and R-Δt MMG-F_END_, R-EMD mainly mechanical components (see text for details); GM, *gastrocnemius medialis*; GL, *gastrocnemius lateralis*; SOL, *soleus*. Data presented as mean(SD).

**Fig 5:**
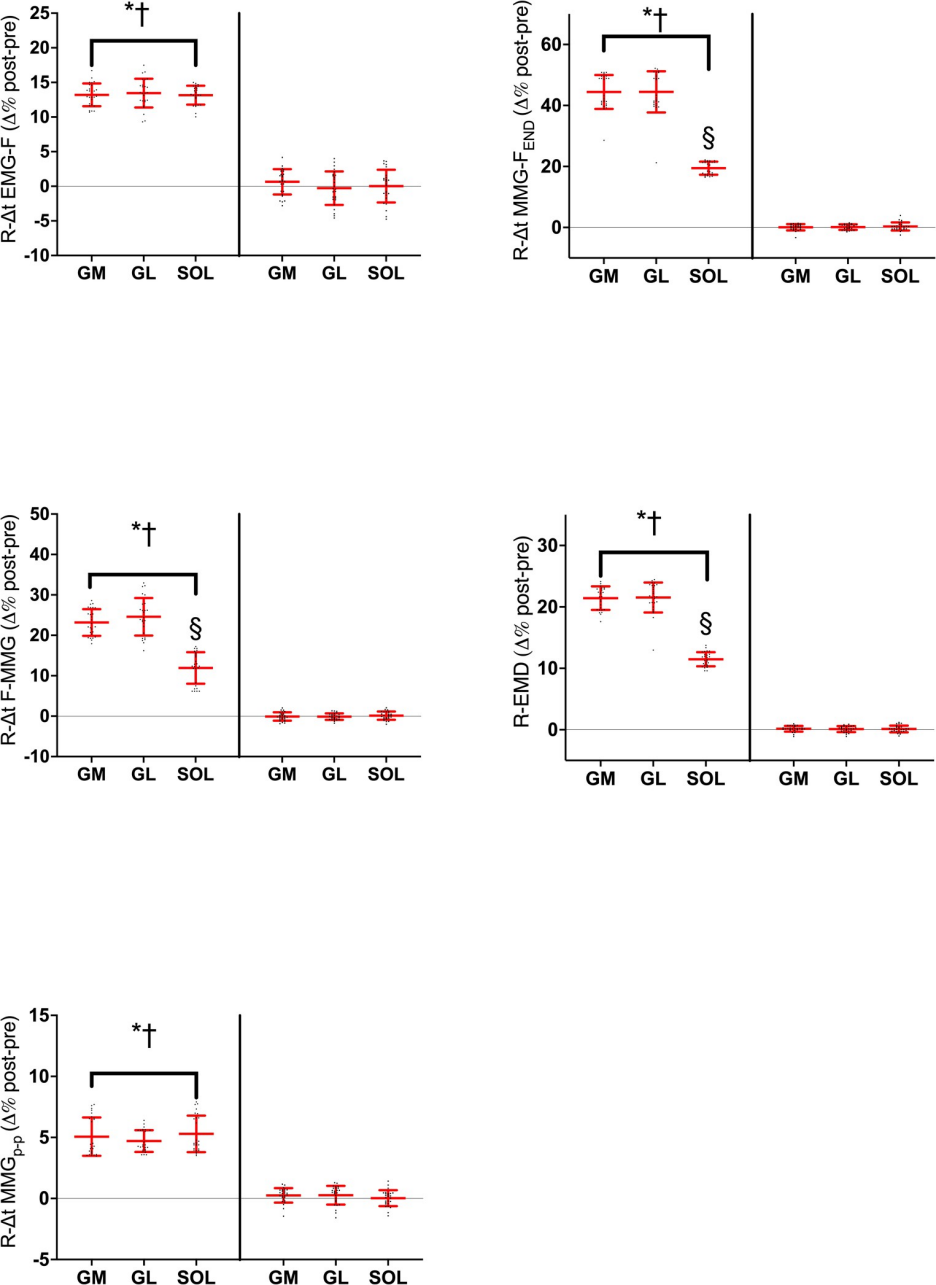
Individual and average post-pre percentage changes in the electromechanical delay components calculated during relaxation (R-EMD) after passive stretching and control in the *gastrocnemius medialis* (GM), *gastrocnemius lateralis* (GL) and *soleus* (SOL). ● = stretched limb; ■ = control; *P<0.05 post *vs*. PRE; †*P*<0.05 *vs*. control; §*P*<0.05 GM and GL *vs* SOL. Horizontal red line represents the average values, the vertical red lines represent the standard deviation. R-EMD, total delay during relaxation phase; R-Δt EMG-F, mainly electrochemical component of R-EMD; R-Δt F-MMG, R-Δt MMG_p-p_, and R-Δt MMG-F_END_, R-EMD mainly mechanical components; see text for details on each component.

The stretch-induced changes in the R-EMD components are provided in Fig 5. Three-way ANOVA found *session* × *time* × *muscle* interaction in R-Δt EMG-F, (*F*_1,2_ = 9.61, *P*<0.001, η_P_^2^ = 0.409) R-Δt F-MMG (*F*_1,2_ = 9.61, *P*<0.001, η_P_^2^ = 0.409), R-Δt MMG-F_END_ (*F*_*1*,*2*_ = 6.30, *P*<0.001, η_P_^2^ = 0.391) and R-EMD (*F*_*1*,*2*_ = 14.21, *P*<0.001, η_P_^2^ = 0.486). At baseline, the average between-muscle in R-Δt F-MMG [44.9(2.1) ms and 45.3(2.7) ms vs. in 40.7(2.2) ms, GM, GL, and SOL, respectively] and in R-EMD [241.1(17.9) ms and 241.5(18.8) ms vs. 231.4(17.8) ms, in GM, GL, and SOL, respectively] were longer in GM and GL than in SOL (*d* from 2.02 to 3.67). Stretching increased all the R-EMD components similarly in all muscles in the stretched muscles (*d* from 0.12 to 4.58). The total increase in R-EMD was longer in GM [21(2) %] and GL [22(2) %], compared to SOL [12(1) %], with *d* ranging from 0.63 to 1.81. Analyzing the different R-EMD components, R-Δt F-MMG [23(3)% and 25(5)% vs. 12(4)%, in GM, GL, and SOL, respectively] and R-Δt MMG-F_END_ [44(6)% ms and 44(7)% vs. 19(2)%, in GM, GL, and SOL, respectively] increased in GM and GL to a greater extent than in SOL (R-Δt F-MMG *d* from 1.25 to 1.96, R-Δt MMG-F_END_ *d* from 0.26 to 2.34). No change occurred in CTRL.

### Correlations

**Fig 6:**
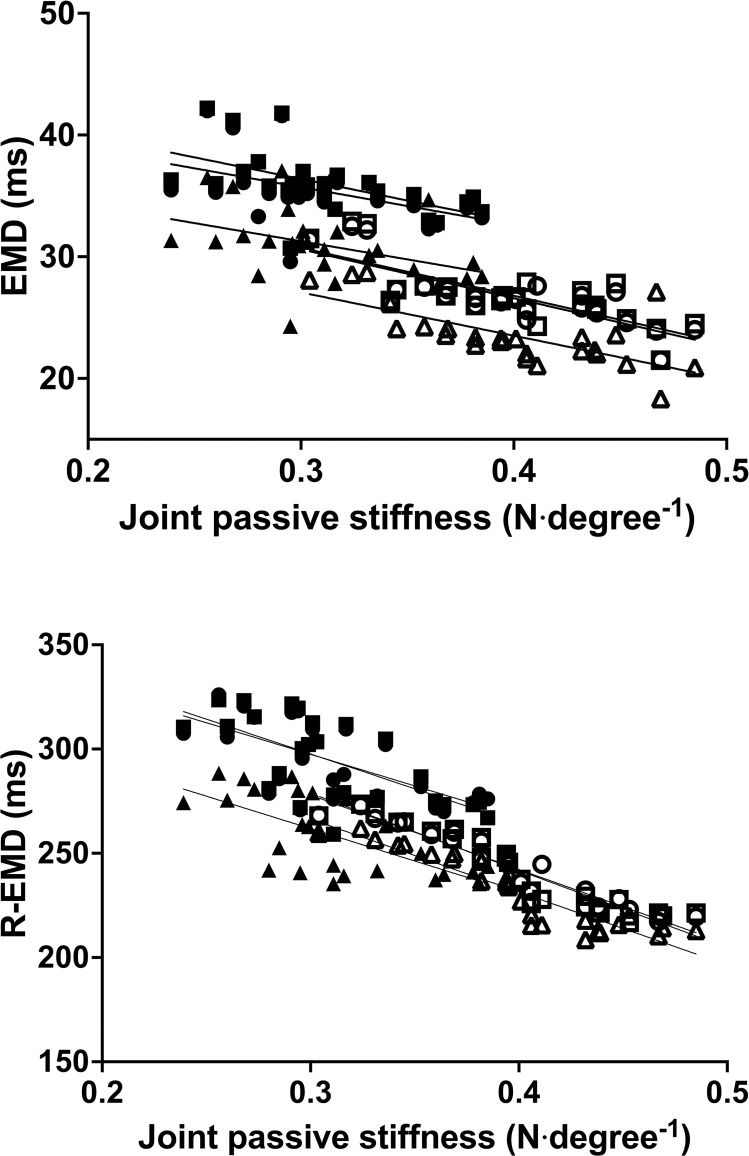
Correlations between joint passive stiffness and during contraction (EMD) and relaxation (R-EMD) in the *gastrocnemius medialis* (GM), *gastrocnemius lateralis* (GL) and *soleus* (SOL) before and after passive stretching. ○ = GM PRE; □ = GL PRE; △ = SOL PRE; ● = GM POST; ■ = GL POST; ▲ = SOL POST.

The correlations between the joint passive stiffness and the EMD and R-EMD for GM, GL, and SOL are reported in Fig 6 and Table 4. With some sporadic exceptions, *moderate*-to-*very high* correlations were found between the joint passive stiffness and the EMD and R-EMD mechanical components in all the three muscles, both at PRE and POST (*r* from -0.477 to -0.926; *P* from 0.007 to <0.001). No correlation was found between the stretch-induced changes in joint passive stiffness and in EMD and R-EMD components (*r* from -0.137 to 0.103; *P*>0.05).

**Table 4:** Correlations between the joint passive stiffness and electromechanical delay during contraction and relaxation phases.

		Contraction phase (n = 26)		Relaxation phase (n = 26)			
Muscle	Joint passive stiffness ↓	Δt EMG-MMG (*r*, *P*)	Δt MMG-F (*r*, *P*)	R-Δt EMG-F (*r*, *P*)	R-Δt F-MMG (*r*, *P*)	R-Δt MMG_p-p_ (*r*, *P*)	R-Δt MMG-F_END_ (*r*, *P*)
GM	PRE	-0.103, 0.698	-0.893, <0.001	-0.361, 0.067	-0.899, <0.001	-0.896, 0.001	-0.926, <0.001
	POST	0.103, 0.683	-0.666, 0.004	-0.299, 0.076	-0.744, 0.002	-0.680, 0.002	-0.543, <0.001
GL	PRE	-0.106, 0.687	-0.760, <0.001	-0.373, 0.051	-0.899, <0.001	-0.886, <0.001,	-0.897, 0.001
	POST	0.103, 0.678	-0.477, <0.001	-0.277, 0.061	-0.569, 0.001	-0.598, 0.002	-0.555, 0.007
SOL	PRE	-0.106, 0.687	-0.760, <0.001	-0.373, 0.051	-0.899, <0.001	-0.886, <0.001,	-0.897, 0.001
	POST	0.103, 0.678	-0.477, <0.001	-0.277, 0.061	-0.569, 0.001	-0.598, 0.002	-0.555, 0.007

EMD and R-EMD, total delay during contraction and relaxation phases, respectively; Δt EMG-MMG and R-Δt EMG-F, mainly electrochemical components of EMD and R-EMD, respectively (see text for details); Δt MMG-F, mainly mechanical EMD component; R-Δt F-MMG, R-Δt MMG_p-p_, and R-Δt MMG-F_END_, R-EMD mainly mechanical components (see text for details); GM, *gastrocnemius medialis*; GL, *gastrocnemius lateralis;* SOL, *Soleus*. For each correlation, Pearson’s product moment correlation coefficient (*r*) and *P*-value are reported.

## Discussion

The present study aimed to assess possible between-muscle differences in the effects of a passive stretching bout on the EMD and R-EMD components, and to determine the correlations between the joint passive stiffness and the EMD and R-EMD mechanical components in the different synergistic muscles, both before and after stretching. The present study adds new information concerning the calculation of the EMD in synergistic muscles. The delays in the quadriceps femoris were previously investigated without underpinning any possible difference between each muscle head [17]. This was due to the use of nerve stimulation that activates synergistic muscles simultaneously [17]. In our current study, we stimulated the motor point of each individual synergistic muscle. Our data revealed larger EMD and R-EMD duration in GM and GL at baseline compared to SOL, mostly attributable to the longer duration of the mainly mechanical component of the EMD and R-EMD (R-Δt F-MMG only). After stretching, we observed a similar lengthening in all EMD components between the synergistic muscles examined. On the contrary, during relaxation GM and GL exhibited larger stretch-induced increase in R-Δt F-MMG and R-Δt MMG-F_END_ compared to SOL. These results suggest that the effect of stretching is more pronounced in the gastrocnemius muscles than SOL. Negative correlations between the joint passive stiffness and the EMD and R-EMD mechanical components were observed for the first time in GL and SOL, and confirmed in GM. The present data support the concept that the stretch-induced prolongation of the contractile response in vivo is correlated with the passive mechanical properties of each synergistic muscles.

### Preliminary considerations

Overall high inter-session reliability and low MDC_95%_ were observed for the dependent variables. The sEMG and MMG signal crosstalk values spanned from 5% to 11%, in agreement with previous literature [27, 36, 37]. Altogether, these results suggest that the EMD and R-EMD components were minimally influenced by crosstalk. Nevertheless, the low but detectable level of crosstalk in peak-to-peak amplitude does not preclude the possibility of current spreading to nearby muscles, which might be responsible for the initial deflection in sEMG and MMG signals.

### Between-muscle differences

The present protocol was conceived to compare the baseline mechanical properties and the stretch-induced responses in three synergistic muscles that could be categorized into biarticular (GM and GL) and monoarticular (SOL) muscles. At baseline, both biarticular muscles showed longer mechanical EMD components during the contraction and longer R-EMD (R-Δt F-MMG) during the relaxation phase compared to SOL. GM and GL have longer tendon compared to SOL, thus increasing the time to strain the tendon during the muscle contraction [5, 6, 25]. Interestingly, GM and GL were reported to have higher prevalence of type-2 muscle fibers than SOL [38]. Since these fibers were shown less stiffer than type-1 muscle fibers, more prevalent in SOL [6], this may have slowed the force transmission in GM and GL. The between-muscle differences were also observed for the effect of stretching, since the R-EMD in GM and GL decreased by higher percentage compared to SOL. These alterations could be induced by the direct strain applied to the muscles that could have reduced the efficiency of the parallel connective tissue such as the endo-, peri- and epimysium to transmit the force to the myotendinous junction and thus to the tendon [6, 25, 39, 40]. While no stretching-induced difference between muscles was observed in the mechanical component of the EMD, during muscle relaxation the R-Δt F-MMG and R-Δt MMG-F_END_ in the GM and GL were greatly lengthened compared to SOL. This is suggestive of slower return of the cross-bridges and the serial elastic components toward their pre-contraction state in GM and GL than in SOL. In partial support of this hypothesis, previous studies [6, 39] claimed that GM may present greater passive tension than GL and SOL at different dorsiflexion angles. Considering the individual characteristics of each muscle, for example the different sarcomere lengths [40] and the different angles of insertion into the Achilles’ tendon [6, 25], these may overall converge into a different initial slack angle, i.e., the joint angle beyond which each muscle begins to develop passive tension [5]. Consequently, when the whole muscle complex is passively stretched, each individual muscle produces different passive tension for a given dorsiflexion angle.

### Correlations between delays and passive stiffness

Previous studies have determined the correlations of joint passive stiffness with the mechanical components of the EMD and R-EMD in GM only [13, 21]. Therefore, the negative correlation of joint passive stiffness with Δt MMG-F during the contraction and with R-Δt F-MMG, R-Δt MMG_p-p_ and R-Δt MMG-F_END_ during the relaxation in GL and SOL are novel in the literature. This is in line with what has been found for GM. On the contrary, no correlation was found between the joint passive stiffness and the mainly electrochemical components of the delays in any muscle. These findings further highlight the role played by the joint passive stiffness in determining the duration of the events included in the mainly mechanical components. Moreover, the correlations found here corroborate the possibility to distinguish the electrochemical from the mechanical components, being the latter much more influenced by the joint stiffness [13]. It should be noted that these correlations were evident when considering the raw data; however, they became much weaker when analyzing the relative changes in the mechanical components of EMD and R-EMD and the changes in stiffness. In addition, considering the upward shift in the relationship between passive stiffness and both EMD and R-EMD following stretching, it is likely that factors unrelated to the reduction in passive stiffness may also contribute to the increase in the mechanical components of EMD and R-EMD [13]. Multiple factors are known to influence the mechanical components of EMD and R-EMD and together with the joint passive stiffness, such as the number of cross-bridges [41], the non-contractile endo- and exo-sarcomeric proteins of the cytoskeleton [42], the parallel connective tissue of the muscle, the diameter of myofibrils, and the mechanical properties of the tendon [43]. Further investigations are required to clarify this aspect.

### Study limitations

Some limitations should be acknowledged. First, we assessed the joint passive stiffness, while more information would come from an examination of the passive stiffness in each individual muscle. Second, we recruited a male sample, while it is known that women have a more compliant muscle-tendon unit than men [44] and different hormone levels that can change the outcomes of the stretching protocol on the connective muscle tissue [45]. Therefore, the present results cannot be extended to different populations.

## Conclusions

By using a combined sEMG, MMG and force approach, we examined the differences in mechanical properties between two biarticular muscles, GM and GL, and a monoarticular muscle, SOL, all synergists and converging into a common tendon. Such an approach allows to investigate the baseline mechanical characteristics of each individual muscle, as well as the individual stretch-induced changes. At baseline, GM and GL showed longer delays during both the muscle contraction and relaxation phases compared to SOL. Additionally, the gastrocnemius was more adversely affected by stretching than the SOL during the relaxation phases. These findings may be explained by inherent differences in tendon length and fiber type of these synergistic muscles [5, 6, 25], as well as a greater susceptibility of the gastrocnemius to stretching. It should be noted that the use of fully extended knee position for both measurements and passive stretching may accentuate these differences. Indeed, previous studies [11, 46] have demonstrated a significant reduction in passive plantar flexion torque and strain in the gastrocnemius muscles, even with a 10–20° degree of knee flexion. Despite these differences, the negative correlation between mechanical delays and the overall joint passive stiffness were observed in all muscles.

## Supporting information

S1 File (XLSX)
